# Influence of TiO_2_ Nanoparticles on the Physical, Mechanical, and Structural Characteristics of Cementitious Composites with Recycled Aggregates

**DOI:** 10.3390/ma17092014

**Published:** 2024-04-25

**Authors:** Carmen Teodora Florean, Horațiu Vermeșan, Timea Gabor, Bogdan Viorel Neamțu, Gyorgy Thalmaier, Andreea Hegyi, Alexandra Csapai, Adrian-Victor Lăzărescu

**Affiliations:** 1 National Institute for Research and Development in Construction, Urban Planning and Sustainable Spatial Development URBAN-INCERC Cluj-Napoca Branch, 117 Calea Florești, 400524 Cluj-Napoca, Romania; carmen.florean@incerc-cluj.ro (C.T.F.); andreea.hegyi@incerc-cluj.ro (A.H.); adrian.lazarescu@incerc-cluj.ro (A.-V.L.); 2 Faculty of Materials and Environmental Engineering, Technical University of Cluj-Napoca, 103-105 Muncii Boulevard, 400641 Cluj-Napoca, Romania; horatiu.vermesan@imadd.utcluj.ro (H.V.); bodan.namtu@stm.utcluj.ro (B.V.N.);

**Keywords:** TiO_2_ nanoparticles, physical–mechanical characteristics, cementitious composites, recycled aggregates, eco-friendly improving technologies, natural aggregates

## Abstract

The aim of this study is to analyze the effect of the addition of TiO_2_ nanoparticles (NTs) on the physical and mechanical properties, as well as the microstructural changes, of cementitious composites containing partially substituted natural aggregates (NAs) with aggregates derived from the following four recycled materials: glass (RGA), brick (RGB), blast-furnace slag (GBA), and recycled textolite waste with WEEE (waste from electrical and electronic equipment) as the primary source (RTA), in line with sustainable construction practices. The research methodology included the following phases: selection and characterization of raw materials, formulation design, experimental preparation and testing of specimens using standardized methods specific to cementitious composite mortars (including determination of apparent density in the hardened state, mechanical strength in compression, flexure, and abrasion, and water absorption by capillarity), and structural analysis using specialized techniques (scanning electron microscopy (SEM) images and energy dispersive X-ray spectroscopy (EDS)). The analysis and interpretation of the results focused primarily on identifying the effects of NT addition on the composites. Results show a decrease in density resulting from replacing NAs with recycled aggregates, particularly in the case of RGB and RTA. Conversely, the introduction of TiO_2_ nanoparticles resulted in a slight increase in density, ranging from 0.2% for RTA to 7.4% for samples containing NAs. Additionally, the introduction of TiO_2_ contributes to improved compressive strength, especially in samples containing RTA, while flexural strength benefits from a 3–4% TiO_2_ addition in all composites. The compressive strength ranged from 35.19 to 70.13 N/mm^2^, while the flexural strength ranged from 8.4 to 10.47 N/mm^2^. The abrasion loss varied between 2.4% and 5.71%, and the water absorption coefficient varied between 0.03 and 0.37 kg/m^2^m^0.5^, the variations being influenced by both the nature of the aggregates and the amount of NTs added. Scanning electron microscopy (SEM) images and energy dispersive X-ray spectroscopy (EDS) analysis showed that TiO_2_ nanoparticles are uniformly distributed in the cementitious composites, mainly forming CSH gel. TiO_2_ nanoparticles act as nucleating agents during early hydration, as confirmed by EDS spectra after curing.

## 1. Introduction

In today’s context, there is intense concern around the world to develop less polluting and more sustainable building materials. The reduction in construction and other waste is also a major concern, as the annual recycling rate of construction waste is less than 5% [1,2]. Therefore, integrating the recycling or reutilization of such waste materials into cementitious construction composites presents a viable strategy to reduce raw material usage and mitigate environmental impact. It is worth noting that these materials encapsulate residual energy from their production phase, further highlighting their potential for sustainability [1,3,4,5,6]. The scientific literature highlights that construction waste consists mainly of concrete, cementitious materials, and ceramic bricks, which account for over 75% of the waste, in addition to a variety of recycled aggregates that are compatible with the cementitious binder matrix [7,8]. Nevertheless, each type of waste, depending on its nature (cementitious or ceramic) and composition, as well as its source of origin, is characterized by several properties (fineness, density, water absorption, mechanical strength, etc.) that will influence the density, water absorption, compressive strength, flexural strength, abrasion resistance, freeze-thaw resistance, or other durability properties of the final cementitious composite [8,9,10,11,12,13,14,15].

Recycled ceramic brick aggregates commonly exhibit higher water absorption rates and lower mechanical strength than natural aggregates, necessitating increased water content during preparation and potentially resulting in reduced mechanical strength of the composite [9,14,16,17]. According to Xue et al. [1], the addition of waste ceramic brick ground to micrometric sizes may initially result in a decrease in the early (7-day) mechanical strengths of cementitious composites. However, when a maximum of 10% ceramic powder is added to the composites, the mechanical strengths at 28 days are higher than those of the control sample (without waste ceramic powder). Yang et al. [9] reported that the use of construction demolition waste containing 14.5% ceramic bricks resulted in a reduction in the workability of the fresh composite, with up to a 20% reduction in compressive strength at 28 days and up to a 34% reduction in tensile splitting strength. Similarly, Cachim [16] demonstrated in his work that the replacement of natural aggregates with waste ceramic brick aggregates reduces the workability of the fresh cementitious composite and the density of the hardened composite but, contrary to most reports, slightly increases the compressive strength of the hardened composite.

The effects of aggregates sourced from recycled glass on cementitious composites are diverse and depend on their granular classification. Thus, the physical–mechanical performance of the composite can be improved by using finer glass powder due to its inherent pozzolanic properties [18]. However, if the waste glass has a larger grain size, it may lead to the development of alkali–silica reactions. This, in turn, can reduce the physical and mechanical performance of the composite [19,20]. Ling and Poon [19] have shown that it is possible to replace even 100% natural aggregates with recycled glass aggregates to produce mortars, provided that the optimum particle size distribution of the recycled aggregates is maintained, with wear resistance being the most obvious improved parameter. According to Ozkan and Yuksel’s study [21], replacing cement with “glass powder” can have apparently contradictory effects: compressive strength increases at low ages for a maximum of 10% replacement but decreases at high ages or for amounts of cement replaced with glass powder greater than 10%.

Incorporating industrial by-products like slag or fly ash in cement formulations frequently leads to a potential decline in early-age mechanical strengths but enhances physical–mechanical performance in later stages, possibly reducing density while enhancing durability [22]. Nevertheless, there is also evidence to suggest that replacing 90% of the sand with slag can result in an increase in capillary water absorption of over 30%, which may have a negative indirect effect on the durability of the composite [23].

Although legislation at the European level has promoted the recycling of waste from electrical and electronic equipment since 2003 [24], there has been limited research to date on the feasibility of incorporating them into cementitious composites. This is mainly due to the significant reduction in physical–mechanical strength caused by these wastes when used as aggregate, which restricts their use to a maximum of 10% as a sand substitute [25,26,27,28]. Some research [25,26,27,28,29,30,31] demonstrated that the replacement of sand with recycled printed circuit board (PCB) waste reduced the compressive strength of the cementitious composite by 38.7% to 95.99% (depending on the substituted NA quantity).

In another area of research, but with a complementary aim of improving the performance of cementitious composites, there are numerous reports in the literature indicating the benefits of using TiO_2_ nanoparticles (NTs) as an admixture material. Thus, TiO_2_ nanoparticles are used to improve some of the physical–mechanical properties of cementitious composites [32]. Additionally, they enable the development of new features such as self-cleaning and hydrophilicity [33,34,35,36,37], increase durability by reducing water absorption, chloride permeability, and CO_2_ permeability, increase resistance to freeze–thaw cycles and sulphate attacks, and reduce the capacity of NO_x_ pollutant compounds [38,39,40,41,42,43,44,45]. Nevertheless, the utilization of TiO_2_ nanoparticles introduces the challenge of altering the rheological properties of the fresh composite owing to their notably extensive specific surface area [35]. Thus, it has been indicated by some authors that the incorporation of 1 wt.% nano-TiO_2_ increased the yield stress and viscosity of pastes by 148% and 24%, respectively [36,37,38,39,40,41]. Studies indicate that the reinforced composite’s performance is improved by expediting cement hydration processes. This is achieved by TiO_2_ nanoparticles acting as nucleating agents primarily in the initial stages of hydration and facilitating the formation of crystalline compounds, notably calcium silicate hydrate (CSH) and ettringite. As a result, densification of the composite matrix occurs, leading to heightened compressive strength along with diminished pore size and their even distribution within the binder mass [41,42,43,44,45,46]. Wang et al. [47] found that replacing 1–5% of cementitious composites with NTs resulted in reduced setting time, increased hydration degree, reduced porosity, and enhanced compressive and flexural strength. Different studies [48,49,50] identified varying optimal percentages of NT replacement, ranging from 1.5% to 10%, with corresponding increases in compressive strength. Regarding flexural strength, there are few reports in the literature, but they indicate an increase of 11% for an optimal amount of 3% NTs [50] and even 51% for an optimal amount of 4% NTs [47]. However, the literature highlights that the use of NTs presents certain challenges, such as identifying the optimal amount of NTs to be added, as excessive quantities may have adverse effects. This is particularly true when it comes to ensuring a homogeneous dispersion of NTs in the composite matrix, as agglomeration can occur, resulting in weak points in the composite [45].

The study of several industrial by-products or recycled materials, such as glass, brick, blast furnace slag, and textolite, as partial replacements for natural aggregates in cementitious composites offers several advantages over focusing on just one material. These materials can be considered aggregates in cementitious composites due to their ability to act as inert filler materials that provide bulk to the composite mixture. Aggregates typically contribute to the mechanical properties of the composite, such as strength and durability, by acting as a framework within the cementitious matrix to distribute loads and resist deformation. While these materials may also have pozzolanic or hydraulic properties, they primarily act as fillers rather than binders, like Portland cement. By replacing some of the natural aggregates with recycled materials, the overall volume of the composite can be maintained, ensuring proper workability and structural integrity [51]. Therefore, considering them as aggregates rather than additives or replacements for Portland cement emphasizes their role in improving the mechanical properties and sustainability of cementitious composites. A comparative analysis of aggregates derived from recycled waste glass (RGA), recycled ceramic brick (RBA), blast furnace slag (GBA), and textolite (RTA) derived from non-metallic waste of electronic equipment, together with the addition of TiO_2_ nanoparticles, allows for the performance of different recycled materials to be assessed in terms of their physical, mechanical, and durability properties [7]. RGA, GBA, and RBA are aggregates derived from abundant sources that have been extensively investigated, yielding numerous findings regarding their potential influences on cementitious composites. In contrast, RTA is a less-explored waste material that lacks thorough documentation of its recycling potential in both the construction sector and various other domains. Previous research suggests that the use of recycled waste aggregates and the addition of TiO_2_ nanoparticles can have varying effects on the properties of cementitious composites, the magnitude of which depends on the amount used and the granularity and type of crystallization. It is important to note that these effects can be both positive and negative. The challenge is to determine the optimum balance to find the most convenient solution for the use of recyclable waste aggregates while exploring the benefits of a low enough amount of TiO_2_ to avoid very high additional costs but high enough to provide mechanical strength and durability benefits for cementitious composites. This comparative approach provides insight into the relative effectiveness of each material and allows the identification of the most suitable options for specific applications where TiO_2_ nanoparticles are to be added. In addition, the study of multiple materials provides a more comprehensive understanding of the challenges and opportunities associated with the incorporation of recycled aggregates and TiO_2_ nanoparticles into cementitious composites. Each material may exhibit unique characteristics, such as varying particle size distributions, chemical compositions, and hydration behavior, which can influence their performance within the composite matrix. Overall, studying the possibility of substituting multiple recycled materials in cementitious composites in conjunction with the addition of TiO_2_ nanoparticles increases the robustness and applicability of research findings, facilitating informed decision-making in the development of sustainable building materials and practices.

Motivated by the above, the aim of this work was to perform an analysis of the influence that the addition of TiO_2_ nanoparticles has on the physical–mechanical performance in correlation with microstructurally induced changes in cementitious composites in which natural aggregates are partially substituted by aggregates derived from recycled waste glass (RGA), recycled ceramic brick (RBA), blast furnace slag (GBA), and textolite (RTA) derived from the non-metallic waste of electronic equipment.

## 2. Materials and Methods

Several types of cementitious composites were designed, prepared, and analyzed for the purpose of the research. Based on the preliminary investigations, which aimed to assess the effect of the incorporation of recycled waste aggregates on the properties of cementitious composites, the current study extends the research initiative with a primary focus on investigating the effects of nano-TiO_2_ addition on composite properties. The preliminary investigations carried out in [52] aimed to determine the optimum proportions of natural aggregates replaced by recycled waste aggregates that would produce the most favorable results in terms of the physical and mechanical properties of the composites. In addition, they highlighted the potential need for additional mixing water, depending on the use of specific types of recycled aggregate. The research hypothesis, formulated from an extensive review of the scientific literature and preliminary results, was that the incorporation of NTs would improve composite properties. However, this improvement followed a Gaussian distribution model, and efforts were focused on identifying the optimum level of NT addition. The control composition (R1) was produced using cement, natural aggregates, a superplasticizer additive, and water. In relation to this control composition, based on preliminary research, compositions (R2–R5) were identified, in which part of the natural aggregates were substituted with aggregates from recycled waste glass, brick, blast furnace slag, or textolite. To analyze the influence of TiO_2_ nanoparticles on the physical–mechanical performance of cementitious composites, in the cement matrixes of the control sample, respective of the composites in which natural aggregates were substituted with recycled waste aggregates, quantities of 2%, 3%, 4%, and 5% TiO_2_ nanoparticles (NTs) were introduced as admixtures, with percentages reported by mass relative to the amount of cement in the composition.

### 2.1. Raw Materials

**Figure 1:**
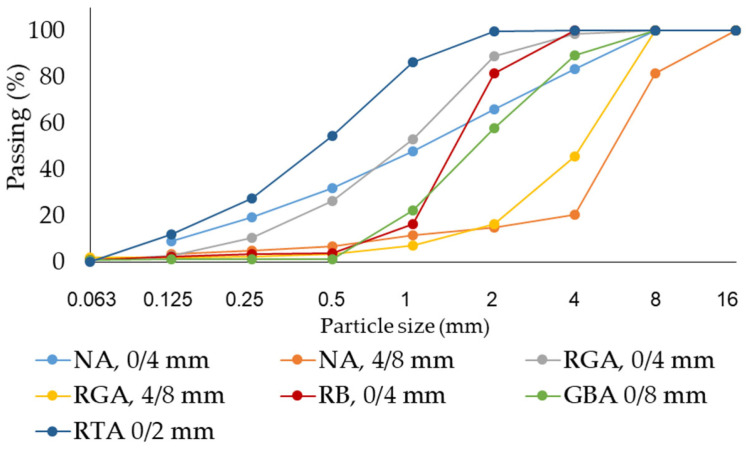
Particle size distribution of the aggregates [52].

**Figure 2:**
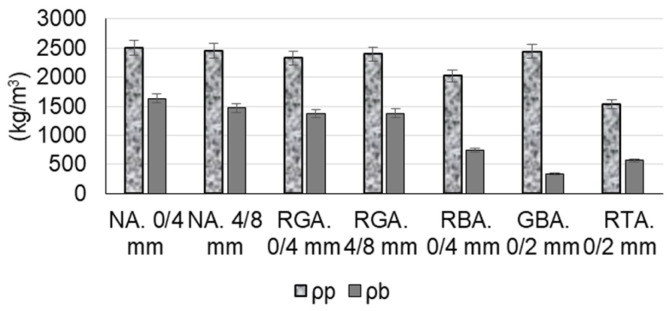
Bulk density (*ρ_b_*) and true density (*ρ_p_*) of the aggregates [52].

**Figure 3:**
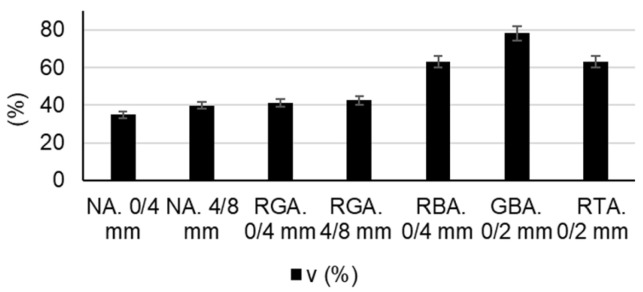
Intergranular bulk porosity (*v*) of the aggregates [52].

**Figure 4:**
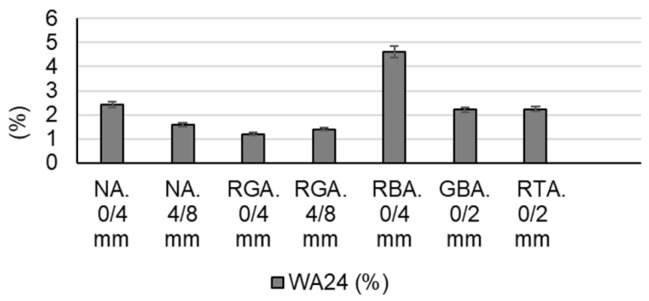
Water absorbtion capacity (*WA*_24_) of the aggregates [52].

The following raw materials were selected for the production of cementitious composites: Portland cement CEM I 52.5 R (HOLCIM Romania, Aleșd, Bihor County, Romania), natural aggregates (NAs) granular class 0/4 mm and 4/8 mm, recycled waste glass aggregates (RGAs) granular class 0/4 mm and 4/8 mm, recycled ceramic brick aggregates (RBAs) granular class 0/4 mm, blast furnace slag (GBA) granular class 0/2 mm, recycled textolite aggregates (RTAs) granular class 0/2 mm, MasterEase 5009 superplasticizer additive (BASF, Ludwigshafen, Germany), water, and TiO_2_ nanoparticles type AEROXIDE^®^ TiO₂ P25 Degussa (Evonik Industries AG, Hanau, Germany). All the recycled waste aggregates were of local origin. The characteristics of the raw materials were as follows:

- Portland cement CEM I 52.5 R was purchased commercially and is characterized by a content of min. 95% Portland clinker and a compressive strength at 28 days of minimum 52.5 N/mm^2^ and maximum 62.5 N/mm^2^.
- MasterEase 5009 superplasticizer/strong water-reducing additive was purchased commercially.
- TiO_2_ nanoparticles of type AEROXIDE^®^ TiO_2_ P25, according to the manufacturer’s technical data sheet, were characterized by a purity of 99.5%, containing more than 70% anatase crystalline phase.
- Natural aggregates as well as aggregates from recycled waste were characterized by determining the particle size distribution curve according to EN 933-1 [53], bulk density and intergranular porosity according to EN 1097-3 [54], and true mass and water absorption coefficient according to EN 1097-6 [55], as shown in Figure 1, Figure 2, Figure 3 and Figure 4. Additionally, for blast furnace slag (GBA), the oxide composition was determined by X-ray fluorescence (XRF) analysis (Table 1). The textolite slag was analyzed for residual metal content (Table 2) according to the methodology indicated in specific standard documents [56,57,58,59].

**Figure 5:**
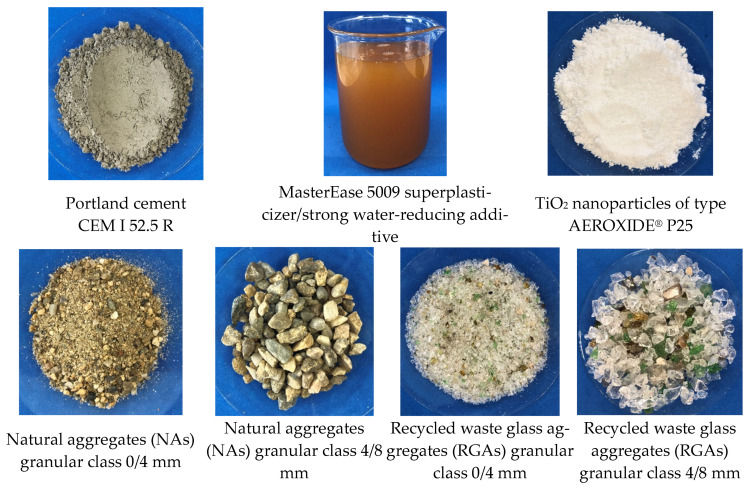

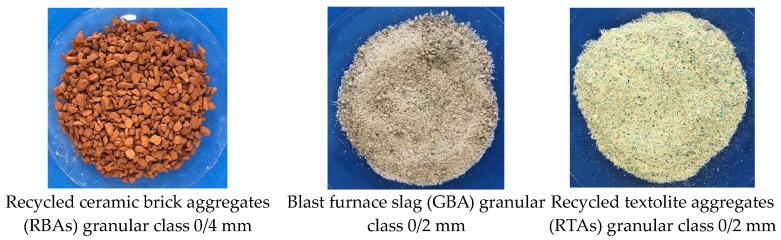
Photographs of the raw materials.

Example images of the raw materials are shown in Figure 5.

### 2.2. Production of Cementitious Composites

The control sample (R1-0) was designed as a Portland cement and natural aggregate-based mortar with a water/cement ratio of 0.6, according to Table 3 [52].

**Figure 6:**
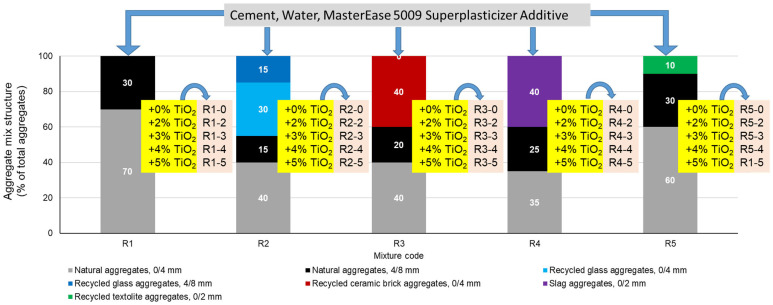
Raw materials used, mixture structure of aggregates, amount of NTs added, and composition identification code for the cement composite mixtures where natural aggregates were substituted with recycled waste aggregates.

Subsequently, for each type of waste proposed to partially replace natural aggregates (glass, brick, slag, or textolite), specific recipes R2-0, R3-0, R4-0, and R5-0 were designed starting from the control sample (R1-0). For these mixtures, the code containing the indicator ‘0’ represents the absence of NTs in the composition. The difference from the control sample is that in the compositions R2-0…R5-0, part of the amount of natural aggregates was replaced by an equal amount of recycled waste aggregates, with all other parameters remaining constant, as shown in Figure 6. The mix-design in which natural aggregates were substituted with recycled waste aggregates (R2-0…R5-0) was based on the condition that each aggregate cumulative distribution curve fell within the favourable range, or at least the usable range, as indicated by the concrete design and preparation standard [60,61,62,63]. Since some of the recycled aggregates used (waste ceramic brick (RBA), slag (GBA), and waste textolite (RTA)) were characterized by higher water absorption than natural aggregates, as shown in Figure 4, in order to not influence the workability of the composites and the cement hydration processes because of insufficient water, prior to preparation, these aggregates were immersed for 24 h in water, then drained, and excess water was removed by swabbing.

In the next step, for each designed mixture in which natural aggregates were substituted by recycled waste aggregates, compositional variants were developed in which 2%, 3%, 4%, or 5% TiO_2_ nanoparticles were added as a mass percentage of the cement quantity. In total, 25 mixtures of cementitious composites were designed and developed. Figure 6 shows the raw materials used, the compositional structure of the aggregates, the amount of NTs added, and the identification code of the compositions according to the types of aggregates and NT content.

For all the compositions prepared, the quantity of cement was kept constant, the quantity of superplasticizer additive used was 0.5%, mass percentages referred to the quantity of cement, and the mixing water was determined considering as a reference indicator the workability expressed by the diameter of the cake on the spreading mass for which the condition of falling within the range 175 ± 10 mm was set. The raw materials were weighed using a KERN FKB 36K0.1 balance (KERN & SOHN GmbH, Balingen, Germany) with an accuracy of 0.1 g.

The preparation of the cementitious composites was achieved by mixing the dry raw materials with water and the MasterEase 5009 superplasticizer additive using an ELE paddle mixer (ELE International Ltd., Milton Keynes, UK).

Based on literature references [36,40,42,46,64,65,66], it is known that NTs are also “consuming” water; therefore, in order not to jeopardize the hydration–hydrolysis reactions of cement due to insufficient water as a result of its absorption by NTs, the amount of water was adjusted so that the workability parameter of the composite remained constant and similar to that of NT-free composites, i.e., the diameter of the cake on the spreading mass, according to EN 196-3 [67], was in the range 175 ± 10 mm. Another difficulty that arose during this stage of the research was ensuring homogeneous dispersion of NTs in the cementitious matrix. Additionally, adjusting the amount of water for preparation proved to be challenging. Due to their tendency to agglomerate and form deposits or islands, which can reduce the yield of benefits induced by photo-activation and even cause negative effects on the physical–mechanical performance of the composite, representing points of vulnerability [7,34,35,36,37,38,39,40,41,42,43,44,45,46,47,48,49,50,51,52,53,54,55,56,57,58,59,64,65,66,68,69,70], a method was chosen for the incorporation of NTs. The raw materials were pre-mixed in the dry phase, and then water and the superplasticizer additive were added.

To maintain the consistency of the fresh composite, based on preliminary tests, it was determined that mixing water supplementation was necessary, both depending on the type of waste used and the amount of NTs added to the mass. Thus, for every 1% NTs added to the composite mass, the mixing water was supplemented by approximately 1.8–3.5 kg water/m^3^ composite, resulting in w/c ratios of w/c = 0.625 for R1-5 and w/c = 0.65 for R5-5, with all other w/c ratio values falling within these limits.

For each casting batch, the specimens were made by casting the fresh composite in metal molds with dimensions 40 × 40 × 160 mm and in metal molds with dimensions 70 × 70 × 70 mm. After casting, the specimens were kept in the molds for 24 h in a humid air box at a constant temperature of 20 ± 1 °C and a relative humidity of min. 90%, then demolded and kept submerged in water at a temperature of 20 ± 2 °C until reaching the age of 28 days.

### 2.3. Analysis of the Physical–Mechanical Performances of Cementitious Composites

The test specimens were tested at the age of 28 days after casting. The following performance indicators were determined and analyzed for each type of cementitious composite: hardened density according to the standard method given in EN 1015-10 [71]; compressive strength and flexural strength according to the standard method given in EN 1015-11 [72] using ADR Auto 250/25 test equipment (ELE International Ltd., Milton Keynes, UK) with an accuracy of 0.01 kN; abrasion wear resistance (Bohme wear) according to the standard method given in EN 1338 [73]; capillary water absorption according to the standard method given in EN 1015-18 [70].

Weighing of specimens was conducted using a KERN FKB 36K0.1 balance (KERN & SOHN GmbH, Balingen, Germany) with an accuracy of 0.1 g and measurement of dimensions using an electronic calliper gauge with an accuracy of 0.01 mm.

Testing was carried out under laboratory conditions of 23 °C and 60% relative air humidity for a series of 3 sets of identical specimens, reporting the result as the arithmetic mean of the individual results.

### 2.4. Microstructural Analysis of Cementitious Composites

The visual macrostructural analysis of cementitious composites was initially conducted without optical magnification equipment to assess the uniformity of aggregate distribution in the cementitious binder matrix. Subsequently, a LEICA SAPO optical microscope (Leica Microsystems, GmbH, Wetzlar, Germany) was used for microscopic analysis to examine the distribution at the macroporosity level.

A JEOL/JSM 5600—LV scanning electron microscope (JEOL Ltd., Tokyo, Japan) operating in the secondary electron imaging (SEI) mode at 15 kV acceleration voltage was used to take SEM and EDS images. As part of the preparation process, the samples were gold coated by plasma sputtering to improve the electrical conductivity for electron microscopy analysis. Phase identification in the samples was carried out using X-ray diffraction (XRD) in the angular range 2θ = 20−85°. An INEL Equinox 3000 diffractometer (Thermo Fisher Scientific S.p.A., Milan, Italy) using Co-Kα radiation (λ = 1.7903 Å) was used.

## 3. Results and Discussions

### 3.1. Influence of the Addition of TiO_2_ Nanoparticles on the Physical–Mechanical Characteristics of Cementitious Composites

**Figure 7:**
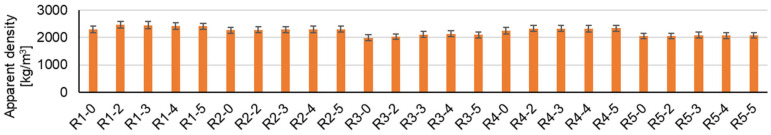
Apparent density.

The experimental results indicate, in terms of the density of cement composites in the hardened state (Figure 7 and Table 4), the following:

- The addition of NTs leads to the densification of the composites. This increase in bulk density occurs in all composites, regardless of whether natural aggregates have been substituted with recycled waste aggregates.
- The most significant increases in bulk density are recorded for the cases where NAs were not substituted with recycled aggregates (7.40–4.66% compared to the sample without NT content, R1-0). However, it can be observed that this increase in density is lower in the case of compositions with a higher addition of NTs.
- The smallest increases in bulk density are recorded for the cases where NAs were substituted with RTAs (0.24–1.41% compared to the NT-free sample, R5-0). This time, it was observed that this increase in density could not be correlated with the amount of NTs introduced as an addition. This non-uniform variation in the parameter could be interpreted as a sign of the inhomogeneity of the distribution of the components, i.e., the difficulty of homogeneous distribution in the composite matrix, on the one hand of RTAs and on the other hand of NTs, concomitant with the inhomogeneity of the formation of cement hydration compounds and pore distribution.
- Regarding the evolution of the parameter for the other recycled aggregate types, in the case of RGAs, there is an increase of 0.88–1.99% compared to the sample without NTs (R2-0); in the case of RBAs, the increase is within 2.11–6.92% compared to the sample without NTs (R3-0); and in the case of GBAs, the increase is within 3.47–3.92% compared to the sample without NTs (R4-0).

**Figure 8:**
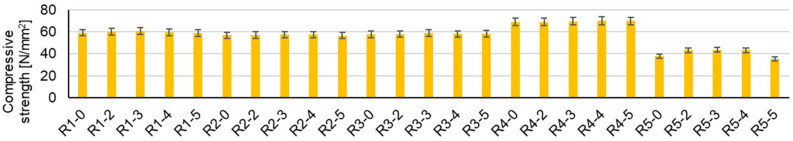
Compressive strength.

**Figure 9:**
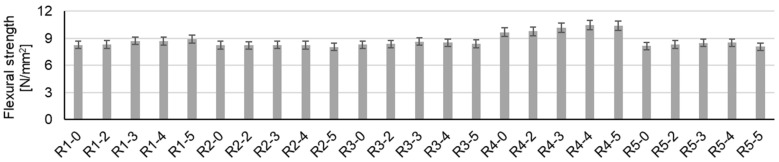
Flexural strength.

In terms of mechanical strengths (compressive strength, Figure 8; flexural strength, Figure 9), for all cementitious composites, with or without substitution of NAs with recycled waste aggregates, a positive effect is observed due to the introduction of NTs into the composite mass (Table 4). This increase in mechanical strength is influenced both by the amount of NTs used as an admixture and by the nature of the aggregates. Thus, in general, an addition of 3% NTs (by mass, relative to the amount of cement) is favorable for increasing the compressive strength (Figure 8) for all types of composites, except for composites in which NAs have been substituted with GBAs, for which the effect is maximum for 4% NTs (by mass, relative to the amount of cement). Overall, the increase in compressive strength due to the introduction of NTs into the composite matrix is not large. Compared to the control samples specific to each type of aggregate (R1-0, R2-0, R3-0, R4-0, or R5-0), this increase in compressive strength shows maximums of: 2.62% for the case of natural aggregate compositions (R1-3); 0.95% for RGA compositions (R2-3); 1.94% for RBA compositions (R3-3); 1.70% for GBA compositions (R4-4); and 16.23% for RTA compositions (R5-3). A significant increase in compressive strength is noted for the RTA compositions. This could be interpreted as an indication of the fact that the nanoparticles present in the binder mass, through their function as nucleating agents and the positive influences they have on the formation of cement hydration compounds, probably manage to contribute significantly to mitigating the negative effect of partial substitution of NAs with RTAs.

Although lower compared to some reports in the literature [43,68,74,75,76,77], which show compressive strength increases of 10% to 80%, depending on test age and NT content (1–4 wt.% NT), it can be said that the research results are in agreement with these reports, maintaining an increasing trend, and in contradiction with those indicated by Shekari and Razzaghi [60], who for a cementitious composite with 1.5% NTs report a reduction in compressive strength of 20.73% at the age of 28 days.

In terms of flexural tensile strength (Figure 9), the results show less obvious improvements with NTs, but in a similar trend to the compressive strength. Thus, the greatest increases in flexural strength can be identified from 8.27 N/mm^2^ (R1-0) to 8.92 N/mm^2^ (R1-5) for NA compositions; from 8.24 N/mm^2^ (R2-0) to 8.28 N/mm^2^ (R2-3) for RGA compositions; from 8.31 N/mm^2^ (R3-0) to 8.64 N/mm^2^ (R3-3) for RBA compositions; from 9.67 N/mm^2^ (R4-0) to 10.47 N/mm^2^ (R4-4) for GBA compositions; and from 8.15 N/mm^2^ (R5-0) to 8.51 N/mm^2^ (R2-4) for RTA compositions. As can be seen, the most favorable situations are also for additions of 3% or 4% NTs (mass percentage, relative to the amount of cement).

**Figure 10:**
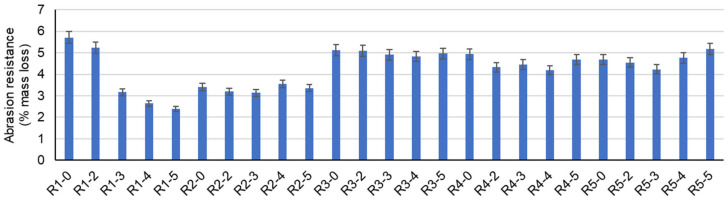
Abrasion resistance.

Abrasion mass loss, the indicator inversely proportional to abrasion resistance (Figure 10 and Table 4), is also influenced by both the nature of the aggregates and the addition of NTs. Thus, in the case of composites with NAs, there is an increasing improvement in abrasion resistance with the increasing addition of NTs, with the best performance for 5% NTs (by mass, relative to the amount of cement). However, this trend is not maintained for the composites in which NAs were partially substituted by recycled waste aggregates, with the most favorable results (lowest mass loss) for 3% or 4% NTs (mass, relative to the amount of cement) as follows: 8.21% decrease in mass loss compared to the control composition (R2-0) for 3% NTs in the composition with RGAs (R2-3); 5.66% decrease in mass loss compared to the control composition (R3-0) for 4% NTs in the composition with RBAs (R3-4); 15% decrease in mass loss compared to the control composition (R4-0) for 4% NTs in the composition with GBAs (R4-4); and 9.62% decrease in mass loss compared to the control composition (R5-0) for 3% NTs in the composition with RTAs (R5-4). A notable phenomenon was observed in the case of composites prepared using only NAs, where wear resistance increased significantly with the increase in the amount of NTs. This observation is consistent with the results reported in the literature and corresponds to the increasing trends observed for compressive and flexural strength and the decreasing trend for water absorption. These results support the hypothesis of matrix densification in the composite as a result of the addition of NTs. Despite the less pronounced improvement trends observed for compressive and flexural strength, it is noteworthy that these behaviors are underpinned by microstructural phenomena such as porosity and microcracking, which are likely to be more pronounced within the bulk rather than the surface layer of the specimen. This surface layer is subjected to abrasion, whereas the entire bulk of the composite is subjected to loading in the case of the other two strength properties.

**Figure 11:**
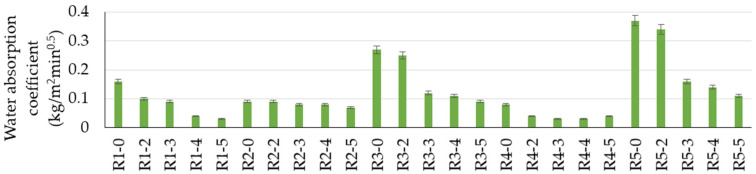
Water absorption coefficient.

From the point of view of water behavior and the coefficient of water absorption by capillarity (Figure 11 and Table 4), it is influenced by both the nature of the aggregates and the addition of NTs. On the one hand, the substitution of natural aggregates with those from waste induces an increase or decrease in water absorption, depending on their nature. Thus, the partial substitution of natural aggregates (NAs) with aggregates from glass waste (RGA) or slag (GBA) leads to an improvement in this performance; the water absorption coefficient by capillarity decreases compared to the composition containing only natural aggregates (R1-0), while the aggregates from ceramic brick waste (RBA) or textolite (RTA) have the reverse effect. With the introduction of TiO_2_ nanoparticles in cementitious compositions, an improvement in this performance is observed for all analyzed cases, more so as the addition is higher. It is also noted that the use of NTs can counteract the negative effects of ceramic brick waste aggregates (RBAs) or textolite (RTA), the most significant reductions in the indicator being recorded in these situations. In the case of brick waste (RBA) or textolite (RTA), it is assessed that 3% NTs can lead to a composition with a water absorption coefficient approximately equal to or even slightly better compared to that of a composition with natural aggregates (0.12 kg/m^2^min^0.5^ for R3-3 and 0.16 kg/m^2^min^0.5^ for R5-3 compared to 0.16 kg/m^2^min^0.5^ for R1-0).

### 3.2. Influence of TiO_2_ Nanoparticle Addition on the Structure and Microstructure of Cementitious Composites

**Figure 12:**
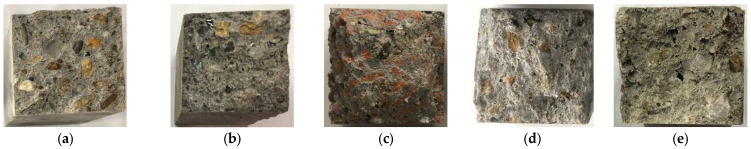
Fracture aspect for cementitious composites made without NTs. (**a**) Cementitious composite with NA; (**b**) Cementitious composite with NA partially substituted with RGA; (**c**) Cementitious composite with NA partially substituted with RBA; (**d**) Cementitious composite with NA partially substituted with GBA; (**e**) Cementitious composite with NA partially substituted with RTA.

**Figure 13:**
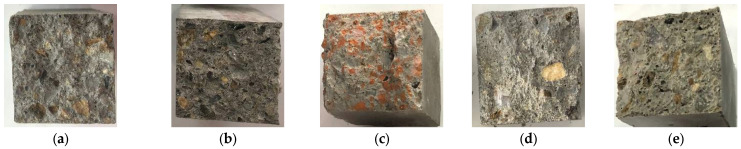
Section appearance of cementitious composites made with NT-exemplary images. (**a**) Cementitious composite with NA, R1-3; (**b**) Cementitious composite with NA partially substituted with RGA, R2-3; (**c**) Cementitious composite with NA partially substituted with RBA, R3-4; (**d**) Cementitious composite with NA partially substituted with GBA, R4-3; (**e**) Cementitious composite with NA partially substituted with RTA, R5-4.

In the case of composites without NTs, visual analysis without optical magnification (Figure 12) indicates a homogeneous distribution of natural aggregates and aggregates from recycled waste in the cementitious binder mass without segregation areas. However, a clear influence of the type of recycled aggregate on the texture of the composite is identified. Thus, the most obvious change in texture is observed in the case of composites with RTAs, where, due to the poor adhesion between the cementitious binder matrix and the recycled aggregate part, the RTA granules are weakly bound, have a random orientation, and have a disorganized appearance in the crack of the composite (Figure 12e). In the case of cementitious composites with 2–5% NT content, the visual analysis records the same homogeneous distribution of aggregates in the binder matrix and the preservation of the specific appearance for the composite with RTA, regardless of the amount of NTs introduced at preparation. Figure 13 exemplifies the appearance of these composites.

**Figure 14:**
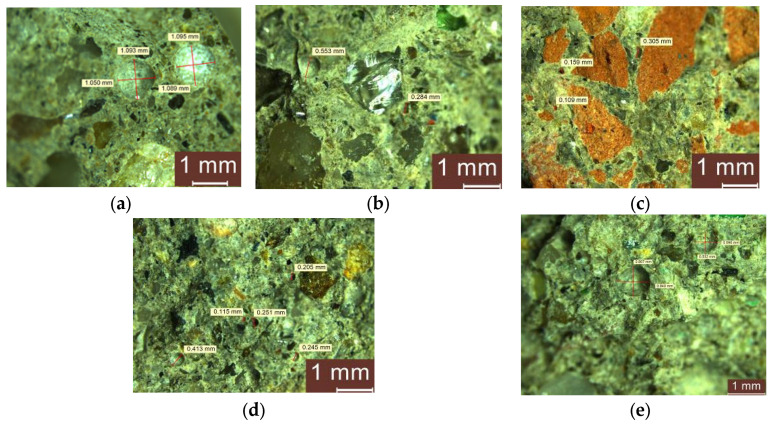
Microscopic analysis of cementitious composites without NTs but (**a**) with NA; (**b**) with NA partially substituted with RGA; (**c**) with NA partially substituted with RBA; (**d**) with NA partially substituted with GBA; and (**e**) with NA partially substituted with RTA.

Optical microscope analysis of NT-free composites, exemplified in Figure 14, highlights the changes that substitution of natural aggregates (NAs) with aggregates from recycled waste induces on the macroporosity of the composites. Thus, for the control composite, pores with dimensions ranging from 0.40–1.1 mm are identified in the binder matrix, with the binder evenly enclosing the aggregate grain. The substitution of NA with RGA causes a numerical increase in the pores simultaneously with the reduction in their dimensions (dimensions generally in the range 0.27–0.60 mm and rarely over 0.8 mm). Additionally, the AN or RGA granules are well embedded in the binder matrix. When RBA is used as a replacement aggregate for NAs, a good incorporation in the binder matrix is observed, with a uniform distribution of pores, more numerous compared to the control, but with smaller dimensions (0.109–0.335 mm). The use of GBA as a substituent aggregate has similar effects, with a uniform distribution of pores in the binder matrix that is more numerous, with dimensions in the range of 0.107–0.500 mm. However, the main difference is observed when RTA is used: at the RTA–cement binder interface, there are numerous areas of no contact, the distribution of RTA in the binder matrix is relatively inhomogeneous, as is the porosity, which is unevenly distributed, and the pore dimensions have large variations, with the diameter reaching almost 1 mm.

**Figure 15:**
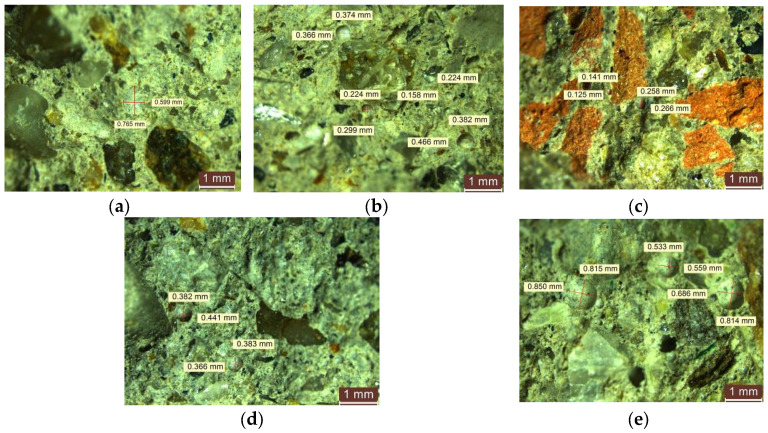
Microscopic analysis of cementitious composites with NTs (**a**) with NA, R1-3; (**b**) with NA partially substituted with RGA, R2-5nt; (**c**) with NA partially substituted with RBA, R3-2nt; (**d**) with NA partially substituted with GBA, R4-2nt; and (**e**) with NA partially substituted with RTA, R5-3NT.

When NTs are introduced into the composite mass, a reduction in pore size is observed in the range of 0.25–0.8 mm for compositions prepared with NAs only and with 2–5% NTs. For composites with 2–5% NT and RGA content, the pore dimensions are in the range of 0.15–0.51 mm; for composites with RBA content, the pore dimensions are in the range of 0.12–0.30 mm; for composites with GBA content, the pore dimensions are in the range of 0.10–0.45 mm; and for composites with RTA content, the pore dimensions are in the range of 0.50–0.85 mm. Example images are shown in Figure 15. Based on these observations, a decrease in pore size can be seen, which is less pronounced in the case of composites with RBA or GBA and more pronounced in the case of composites with NA, RGA, and RTA.

**Figure 16:**
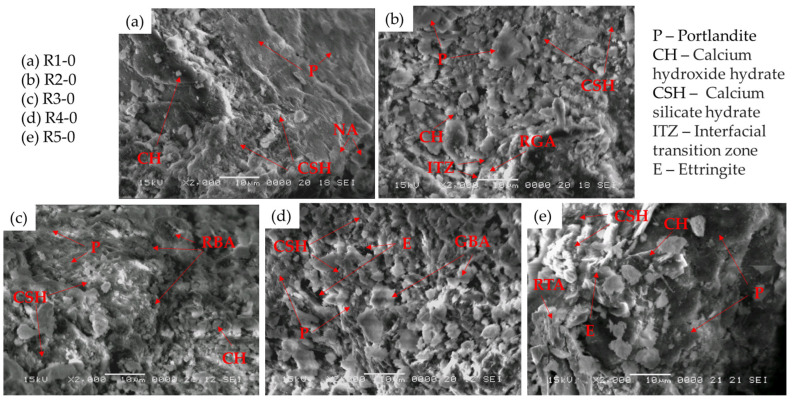
SEM images of the samples without NTs: (**a**) NA sample R1-0; (**b**) Cementitious composite with NA partially substituted with RGA, R2-0; (**c**) Cementitious composite with NA partially substituted with RBA, R3-0; (**d**) Cementitious composite with NA partially substituted with GBA, R4-0; (**e**) Cementitious composite with NA partially substituted with RTA, R5-0.

Figure 16 depicts the SEM images of the cementitious composite with NA (Figure 16a) and the cementitious composites with NA partially substituted with RGA (Figure 16b), RBA (Figure 16c), GBA (Figure 16d), and RTA (Figure 16e).

The compatibility of the natural aggregate, as well as RBA, GBA, and RTA, with the cement is shown to be cohesive, forming a coalesced mass with no visible cracks between the aggregates and the cement matrix. However, in the case of the sample containing RGA, there is a distinct interfacial transition zone (ITZ) between the aggregate surface and the cement matrix, indicating a weaker compatibility between RGA and the cement paste, as confirmed by the slight decrease in flexural strength and compressive strength. The SEM images indicate that the NA, RBA, and RTA samples present a finer pore structure than those containing RGA and GBA. The main hydration products observable for all samples are the rod-like crystals of ettringite (E), the calcium hydroxide hydrate (CH), and the calcium silicate hydrate (CSH), a highly porous, poorly crystalline structure. Ettringite precipitation occurs during the hydration process of Portland cement when calcium aluminate reacts with calcium sulfate, and its presence depends on the ratio of tricalcium aluminate. Variations in the ettringite ratio, correlated with its permeability to water and interaction with other chemical species, have a significant impact on the structural progression during cement hydration, which in turn affects the resulting properties of the cement. Ettringite formation, which is critical in determining the setting rate of the highly reactive aluminate phases, dominates the initial phase of cement hydration. A direct result of this process is volume expansion in fresh concrete, which has been proven to cause cracking in cured concrete during events such as delayed ettringite formation (DEF) and sulfate attack cement degradation [61].

In Figure 16a, an SEM micrograph illustrates the cementitious composite with natural aggregates (R1-0). The image shows a heterogeneous material where portlandite dominates the surface and emerges as the primary product after 28 days of concrete curing. As a result of the consumption of portlandite in the pozzolanic reaction, areas rich in CSH can be observed in the image. The RGA sample (Figure 16b) shows a relatively dense structure with no apparent cracks or pores. Extensive regions of CSH and portlandite cover most of the surface, leaving only a thin region representing the interfacial transition zone between the aggregate particle and the cement matrix. The glass granules, with sizes of 0/4 mm and 4/8 mm, are evenly dispersed within the concrete matrix. However, due to the presence of sharp particle edges and a smooth matrix surface, the possibility of insufficient bonding between the cement mortar and glass particles arises. Furthermore, the formation of CSH gel in aggregated networks with relatively small pores, combined with the limited water absorption capacity of the glass (in contrast to fine glass powder used as a concrete replacement in previous studies [62,63]), resulted in a small decrease in compressive strength.

Similarly, Figure 16c–e illustrates the morphology of the RBA, GBA, and RTA samples. There is no discernible ITZ for the RBA and GBA samples; however, an ITZ is evident in the sample containing RTA. The RBA has a porous structure with a rough, irregular surface, which greatly enhances the interlocking between the two surfaces. The compactness of the sample can also be attributed to the pozzolanic reaction between the RBA and the Ca(OH)_2_ crystals, facilitated by the absorption of free water in the concrete resulting from the introduction of RBA as well. This reaction likely contributed to a decrease in the porosity of the hardened matrix, consequently leading to an increase in the overall flexural strength. In the sample containing GBA, the microstructure reveals the presence of several characteristic mineral phases. Larger pores with ettringite needles are distributed throughout the matrix, and the surface is predominantly covered by reticulated CSH gel. In addition, small areas show the presence of portlandite crystals. Angular particles of GBA are scattered throughout the microstructure, indicating incomplete hydration of the slag. This incomplete hydration is attributed to the lower reaction activity of the slag compared to that of the Portland cement. However, it is the foil-like, dense, crack-free, amorphous gel structure that provides the benefits of slag in increasing the compressive and flexural strength of composites. With respect to the RTA sample, the microstructure shows a dense composition with portlandite as the dominant mineral phase. Notable hydration products include ettringite and CSH gel. Thicker rod-like structures corresponding to the RTA are observed in the microstructure and appear to be well embedded in the cementitious matrix. However, the adhesion between the sleek surface of RTA and the smooth cementitious matrix is limited. This, coupled with the absence of any pozzolanic effect from RTA, has resulted in a significant reduction in compressive strength and a notable decrease in flexural strength.

**Figure 17:**
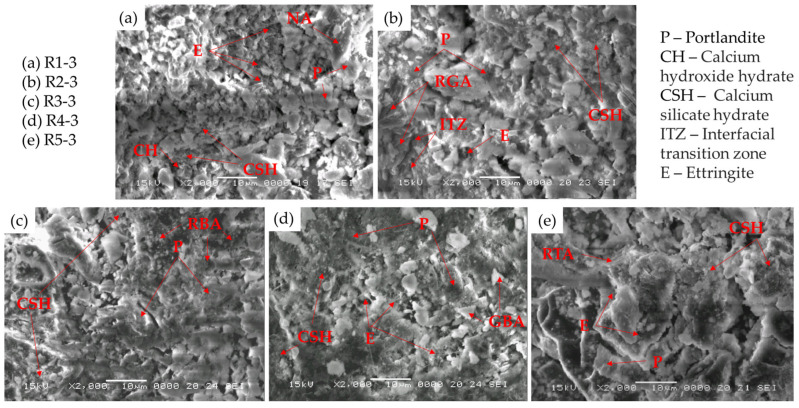
SEM images of the samples containing NTs: (**a**) NA sample R1-3; (**b**) Cementitious composite with NA partially substituted with RGA, R2-3; (**c**) Cementitious composite with NA partially substituted with RBA, R3-3; (**d**) Cementitious composite with NA partially substituted with GBA, R4-3; (**e**) Cementitious composite with NA partially substituted with RTA, R5-3.

The SEM micrographs of samples R1-3, R2-3, R3-3, R4-3, and R5-3 after 28 days of curing are shown in Figure 17. In the NA sample containing 3% NTs (Figure 17a), the microstructure consists mainly of reticulated CSH gel, with only small areas of portlandite visible. This phenomenon can be attributed to the TiO_2_ particles acting as nucleating agents, particularly in the early stages of the hydration process, thereby accelerating cement hydration and promoting the formation of crystalline compounds. A similar trend is observed for the RGA, RBA, GBA, and RTA samples. In the case of the RGA sample (Figure 17b), the ITZ is less pronounced, with RGA particles exhibiting greater cohesion with the cementitious matrix. Pores containing needle-shaped ettringites are scattered in areas of RGA. Figure 17c shows the sample containing RGA + 3% NTs (R3-3), where portlandite crystals are rarely observed in the analyzed area and CSH gel appears as the predominant hydration product with no discernible ITZ.

Within the BGA microstructure (Figure 17d), numerous large pores filled with ettringite (3CaO-Al_2_O_3_-3CaSO_4_-32H_2_O) can be observed, indicating a similar hydration mechanism between GBA and Portland cement. Unreacted GBA particles, together with portlandite crystals, can be identified throughout the investigated area. In comparison, the RTA sample (Figure 17e) shows only a few areas of ettringite. CSH emerges as the predominant hydration product, with small clusters of portlandite identified throughout the observed section. The rod-shaped RTA particles are sparse and barely discernible within the cementitious matrix due to the extensive coverage by CSH, influenced by the presence of TiO_2_ nanoparticles during the hydration process.

The prevalent presence of CSH gel as the primary hydration product is observed, exhibiting reduced porosity compared to images of samples lacking NTs, showcasing a dense, sheet-like structure spanning considerable surface areas. The absence of distinct regions featuring portlandite, which typically exhibits lower elastic modulus and hardness, alongside the emergence of ettringite likely contributed to the enhanced compressive and flexural strength observed in the majority of samples following NT incorporation. Moreover, the increase in abrasion resistance of the samples containing NTs can be explained by their higher compressive and flexural strengths.

**Figure 18:**
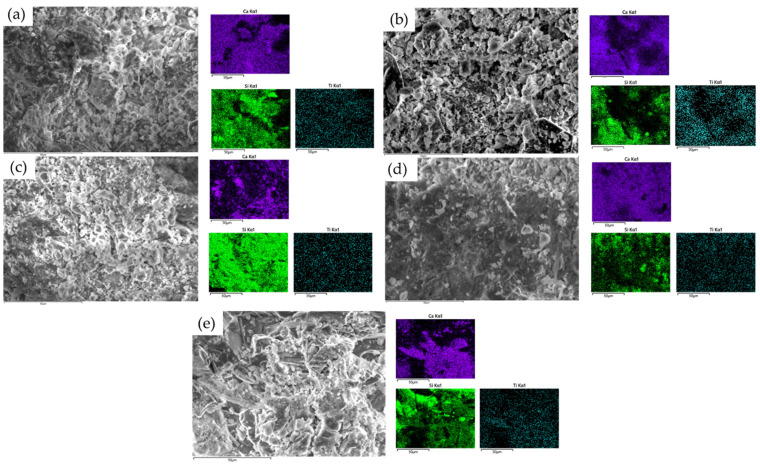
SEM and EDX images of the samples containing (**a**) NTs and 3% NTs (R1-3); (**b**) NA partially substituted with RGA + 3% NTs (R2-3); (**c**) NA partially substituted with RBA + 3% NTs (R3-3); (**d**) NA partially substituted with BGA + 3% NTs (R4-3); (**e**) NA partially substituted with RTA + 3% NTs (R5-3).

Figure 18 displays the EDX spectra of samples, including (a) NTs and 3% NTs (R1-3); (b) NA partially substituted with RGA + 3% NTs (R2-3); (c) NA partially substituted with RBA + 3% NTs (R3-3); (d) NA partially substituted with BGA + 3% NTs (R4-3); and (e) NA partially substituted with RTA + 3% NTs (R5-3). The spectra were recorded at different selected punctual areas to gather additional information regarding the elemental composition of specific regions. The distribution of three different elements, respectively, Ca, Si, and Ti, has been considered in all samples. Therefore, it can be seen that Ca is evenly distributed in all the R1-3 to R5-3 samples over the whole examined area. In the sample containing NA + 3% NTs (Figure 18a), Si is evenly distributed over the area examined. However, in the sample containing RGA (Figure 18b), Si appears to be preferentially distributed in certain locations due to the higher content resulting from the presence of recycled glass aggregates. Similar to R1-3, the sample containing RGB + 3% NTs (Figure 18c) shows an area of uniform Si distribution, whereas for the samples containing GBA + 3% NTs and RTA + 3% NTs, the distribution of Si appears to be less dense and more localized to specific areas of the sample. Ti appears to be uniformly scattered in all samples, suggesting a homogeneous distribution of TiO_2_ in the cementitious composite.

**Figure 19:**
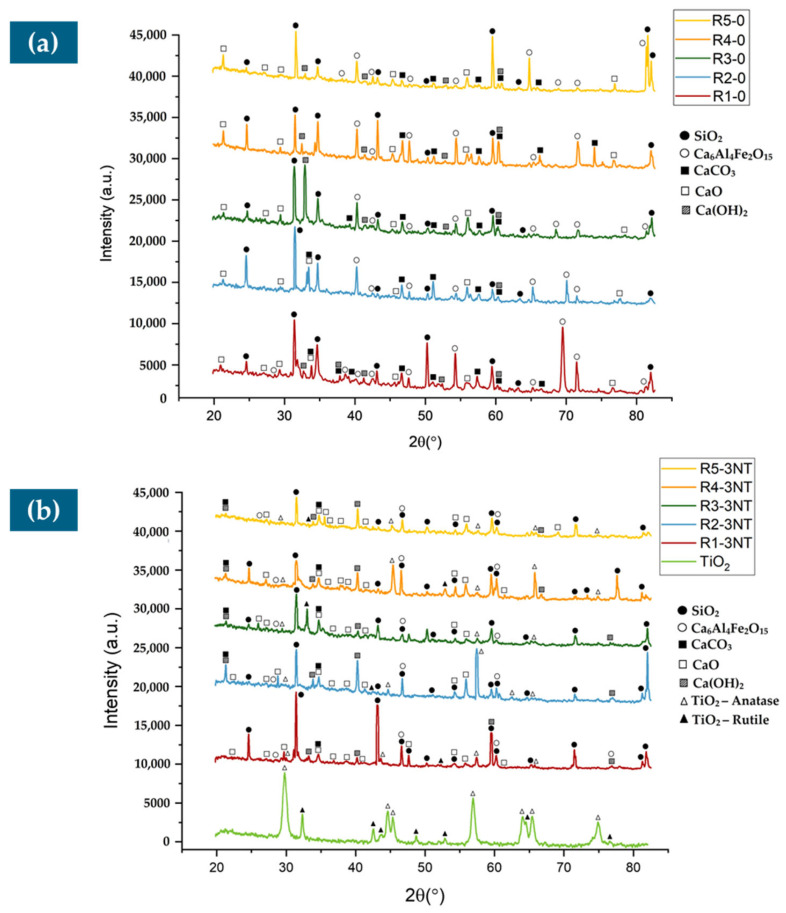
XRD patterns of the (**a**) samples R1-0 to R5-0, without the NTs; (**b**) samples R1-3 to R5-3, with NTs.

Figure 19 presents the comparison XRD patterns of the samples without NTs, R1-0 to R5-0 (Figure 19a), and the samples containing 3% NTs, R1-3 to R5-3 (Figure 19b). Figure 19a reveals the presence of primary peaks characteristic of ordinary Portland cement, such as calcite—CaCO_3_ (present at 2θ = 25.2°, 31.5°, and 33.9°), aluminoferrite—Ca_6_Al_4_Fe_2_O_15_ (present at 2θ = 27.9°, 38.1°, and 55.4°), and portlandite—Ca(OH)_2_ (present at 2θ = 21.0°, and 40.2°). In addition, Figure 19b presents the XRD pattern for the anatase and rutile phases of the TiO_2_ nanoparticles. As it can be observed in the spectra for the samples R1-3 to R5-3, the presence of peaks specific for the anatase phase of the nano-TiO_2_ particles is noticeable at 2θ = 29.6°, 43.1°, 57.2°, and 64.1°. Several peaks corresponding to the rutile phase of the TiO_2_ nanoparticles can be observed in different spectra at 2θ = 32.5° for R3-3 and R5-3, 42.9° for R2-3, and 53.7° for R1-3 and R4-3. This underlines the lack of chemical interaction between the TiO_2_ nanoparticles and the cementitious matrix and the clear presence of a predominantly anatase phase in the samples containing 3% NTs.

Summarizing the experimental results, it can be concluded that the incorporation of NTs improves the densification of the cementitious composite matrix, as demonstrated by both the physical–mechanical parameters and the microstructural investigations, potentially heralding an improvement in durability. However, an overarching observation is that, while the properties of the aggregates used significantly enhance the physical–mechanical performance, the optimum percentage of NTs to maximize the benefits varies from case to case. The increase in matrix densification is most strongly supported by the variation in water absorption coefficient and the progression of compressive, flexural, and abrasion strengths, whereas the influence on apparent density is less pronounced. This phenomenon is likely to be due to localized microstructural changes in porosity, as evidenced by SEM and EDS analyses, which pose a challenge in quantifying their representation in the high values expressed for apparent density.

## 4. Conclusions

The study of recycled materials such as glass, brick, blast furnace slag, and textolite as partial replacements for natural aggregates in cementitious composites offers several advantages, including improved mechanical properties and sustainability. By analyzing these materials alongside TiO_2_ nanoparticles, insights into their physical, mechanical, and durability properties can be gained, facilitating informed decision-making in sustainable construction practices.

- The introduction of NTs into the composite mass, regardless of the type of aggregates used, has the effect of increasing the apparent density in the hardened state.
- The introduction of NTs into the composite mass, regardless of the type of aggregates used, has the effect of increasing the compressive strength, with a maximum obtained for 3% or 4% NTs, with values between 43.3 and 70.13 N/mm^2^, depending on the nature of the recycled aggregates substituted for the natural aggregates.
- The introduction of NTs into the composite mass, regardless of the type of aggregates used, has the effect of increasing the flexural strength, with a maximum obtained for 3% or 4% NTs, with values between 8.24 and 10.47 N/mm^2^, depending on the nature of the recycled aggregates substituted for the natural aggregates.
- The introduction of NTs into the composite mass, regardless of the type of aggregates used, has the effect of increasing wear resistance, with a maximum obtained for 3% or 4% NTs, with values between 0.03 and 0.11 kg/m^2^min^0.5^, depending on the nature of the recycled aggregates substituted for the natural aggregates.
- The introduction of NTs into the composite mass, regardless of the type of aggregates used, has the effect of reducing water absorption by capillarity. In some cases, if recycled aggregates replacing natural aggregates induce an effect of increasing co-efficient water absorption, adding NTs can reduce or even cancel this effect.
- The SEM micrographs of samples R1-3, R2-3, R3-3, R4-3, and R5-3 after 28 days show different microstructures, such as a predominantly reticulated CSH gel in the NA sample with 3% NTs, attributed to TiO_2_ acting as a nucleating agent, accelerating cement hydration; a similar trend is observed for RGA, RBA, GBA, and RTA samples, with variations in ITZ, cohesion, and predominant hydration products, highlighting the strong influence of TiO_2_ nanoparticles on the hydration process. The EDX spectra complete the TiO_2_ nanoparticle distribution image across the investigated areas, confirming the even distribution of the nanoparticles across the sample and therefore indicating a homogenous distribution of TiO_2_ in the cementitious composites.
- The XRD patterns of the samples with and without NTs reveal characteristic peaks of ordinary Portland cement (such as peaks corresponding to calcite, aluminoferrite, and portlandite) and distinct peaks for anatase and rutile phases of TiO_2_ nanoparticles in the samples R1-3 to R5-3, indicating a lack of chemical interaction with the cementitious matrix and the prevalence of the anatase phase in 3% NT-containing samples.

In conclusion, this study serves as a solid foundation for future research efforts aimed at advancing waste recycling by incorporating it into innovative cementitious matrices alongside the development of highly durable materials. Such initiatives hold great promise for environmental conservation and improved structural resilience. A first avenue for such material advances could be the production of small-scale prefabricated components (e.g., paving elements, cladding units, decorative and finishing elements, urban furniture) made from cementitious composites with a blend of natural aggregates partially replaced by recycled counterparts, complemented by a surface wear layer made from a cementitious composite reinforced with NTs to increase durability.

## Figures and Tables

**Table 1:** Characterization of blast furnace slag (GBA) [52].

	SiO_2_	Al_2_O_3_	Fe_2_O_3_	CaO	MgO	SO_3_	Na_2_O	K_2_O	P_2_O_5_	TiO_2_	Cr_2_O_3_	Mn_2_O_3_	P.C.
w%	30.20	10.05	14.70	37.40	4.05	-	0.20	0.38	-	<0.52	<0.05	2.15	-

**Table 2:** Characterization of waste textolite (RTA) [52].

	As	Ba	Cd	Cr	Cu	Hg	Mo	Ni	Pb	Sb	Se	Zn
mg/kg	2.85	603.8	<0.03	18.2	1064	<0.003	<3.0	<0.20	<0.30	<0.5	<0.015	12.59

Note: the “<“ sign represents values below the detection limit of the method.

**Table 3:** Control sample mixture (R1-0).

Mixture Code	Design Class	Water/ Cement Ratio	Cement (kg/m^3^)	Natural Aggregates, Cumulative (kg/m^3^)	Natural Aggregates, 0/4 mm (% of Total Aggregates)	Natural Aggregates, 4/8 mm (% of Total Aggregates)	MasterEase 5009 Superplasticizer Additive (% Mass Ratio to Cement Quantity)
R1	C 20/25	0.6	366	1577	70	30	0.5

**Table 4:** The average values of the experimental results.

Code	w/c Ratio	Apparent Density (kg/m^3^)	Flexural Strength (N/mm^2^)	Compressive Strength (N/mm^2^)	Abrasion Resistance (% Mass Loss)	Coefficient of Water Absorption by Capillarity (kg/m^2^min^0.5^)
R1-0	0.60	2298	8.27	59.18	5.71	0.16
R1-2	0.61	2468	8.32	60.03	5.24	0.10
R1-3	0.62	2453	8.72	60.73	3.16	0.09
R1-4	0.62	2411	8.71	59.38	2.64	0.04
R1-5	0.63	2405	8.92	59.04	2.40	0.03
R2-0	0.60	2262	8.24	56.93	3.41	0.09
R2-2	0.61	2282	8.22	56.98	3.19	0.09
R2-3	0.62	2287	8.28	57.47	3.13	0.08
R2-4	0.62	2291	8.24	57.43	3.56	0.08
R2-5	0.63	2307	8.03	56.47	3.36	0.07
R3-0	0.61	1993	8.31	57.75	5.12	0.27
R3-2	0.62	2035	8.38	57.79	5.09	0.25
R3-3	0.63	2111	8.64	58.87	4.91	0.12
R3-4	0.64	2131	8.51	58.04	4.83	0.11
R3-5	0.64	2095	8.40	58.23	4.96	0.09
R4-0	0.61	2246	9.67	68.96	4.93	0.08
R4-2	0.62	2332	9.78	69.01	4.33	0.04
R4-3	0.63	2331	10.17	69.55	4.45	0.03
R4-4	0.64	2324	10.47	70.13	4.19	0.03
R4-5	0.65	2334	10.40	69.88	4.68	0.04
R5-0	0.62	2054	8.15	37.58	4.68	0.37
R5-2	0.64	2059	8.32	43.31	4.55	0.34
R5-3	0.64	2083	8.50	43.68	4.23	0.16
R5-4	0.65	2072	8.51	43.30	4.76	0.14
R5-5	0.65	2077	8.06	35.19	5.17	0.11

## Data Availability

Data are contained within the article.
